# Intergenerational support and older adults mental health in rural China: evidence and policy implications

**DOI:** 10.3389/fpubh.2026.1780185

**Published:** 2026-05-11

**Authors:** Zhixuan Li, Yongchang Wu, Shan Jin, Jing Chen

**Affiliations:** 1 Institute of Agricultural Economics and Development, Chinese Academy of Agricultural Sciences, Beijing, China; 2 Department of Land and Property Management, Royal Agricultural University, Cirencester, United Kingdom

**Keywords:** child, intergenerational support, mental health, parent, rural older adults

## Abstract

**Introduction:**

Mental health problems among the rural older adults pose a major challenge in contemporary China, where adult children have a crucial role in addressing this issue. However, the mental health experiences of rural older adults remain comparatively underexplored in existing research, particularly in contrast with their urban counterparts.

**Methods:**

Data were collected through face-to-face surveys with 975 adults aged 60 and over from two rural counties with distinct socioeconomic contexts: Kunshan in Jiangsu Province (an economically developed population inflow area, *N* = 431) and Yudu in Jiangxi Province (a typical central China population outflow area, *N* = 544). This design enabled examination of intergenerational support dynamics across different migration contexts.

**Results:**

Intergenerational support, especially economic (*β* = 0.1819, *p* < 0.1) and emotional support (*β* = 0.6427, *p* < 0.01), significantly enhances the mental health of rural older adults, both directly and by influencing perceived intergenerational intimacy. Yet, adherence to the traditional value of “raising children for old-age support” weakens this positive effect by instrumentalizing parent–child relationships. Moreover, the effects of intergenerational support on mental health vary across regions and between genders.

**Discussion:**

The need for policies that not only account for regional and gender differences but also challenge traditional values of child-based old-age support to better promote the mental wellbeing of rural older adults in China.

## 1 Introduction

In China, rapid population aging represents a serious challenge to public health, including mental health among the older adults. By the end of 2024, China’s population aged 60 and above reached 310.31 million, accounting for 22.0% of the total population; those aged 65 and above numbered 220.23 million, representing 15.6% of the total population (72). Loneliness and social isolation are key risk factors for mental health conditions in later life, with 26.4% of the older adults exhibiting depressive symptoms (1). In recent years, the Chinese government has paid increasing attention to mental health issues among older adults. This policy shift is reflected in key national documents such as the *Healthy China 2030* Planning Outline and the *15th Five-Year Plan for National Economic and Social Development*, which explicitly prioritize mental health services for older adults. The *15th Five-Year Plan* proposes to “strengthen mental health and psychological services” and to “enhance early detection and comprehensive intervention for common mental disorders and psychological problems among key populations.” As part of these efforts, from June 23 to 29, 2025, the National Health Commission (NHC) and the National Administration of Traditional Chinese Medicine (NATCM) organized the 2025 National Older Adults Health Promotion Week, with a focus on mental health (2). Nonetheless, a pronounced disparity in healthcare resources between urban and rural areas has received little attention in national policy initiatives (3). Evidence from a systematic review and meta-analysis indicates that suicide rates among older adults in rural China are significantly higher than those in urban areas (4). Accordingly, the mental health challenges faced by older adults in rural settings, where healthcare provision remains comparatively limited, warrant closer examination to inform the development of more context-sensitive and targeted policies.

A growing body of research has demonstrated that older adults mental health is shaped by a combination of individual factors and socioeconomic, cultural, and environmental contexts in which older adults live (2, 5–12). For example, individuals with lower household income tend to report a higher prevalence of depression and anxiety symptoms, greater loneliness, and lower overall wellbeing than those with higher income (13). Cognitive impairments have also been found to predispose individuals to depressive episodes (10). Moreover, perceived upward mobility, whether intergenerational, intragenerational, or prospective, is positively associated with better mental health outcomes (6). Adult children constitute an important part of older adults’ socioeconomic circumstances, for example, by providing their parents with financial, instrumental, and emotional support. However, the mental health consequences of such child-to-parent intergenerational support are not always consistent. Some scholars contend that financial support from children enhances older adults’ subjective wellbeing (14, 15), whereas others argue that reliance on children for financial assistance can adversely affect mental health (16). Similar mixed findings have been reported regarding the effects of instrumental and emotional support (14, 17). Importantly, the mental health experiences of rural adults remain relatively underexplored compared to urban adults in existing research (18).

To address this knowledge gap, the present study investigates how child-to-parent intergenerational support influences the mental health of older adults in rural China. This focus is particularly important in light of the large-scale migration of younger rural populations to urban areas over the past 40 years, which has left many older adults behind and potentially exacerbated experiences of loneliness (19). To capture socioeconomic and cultural variation, we selected Kunshan City, a county-level city in Jiangsu Province, and Yudu County in Jiangxi Province as case study sites. By examining the role of intergenerational support in shaping mental health outcomes, this study contributes to the development of interventions aimed at improving the mental wellbeing of rural older adults, while also addressing regional disparities.

The remainder of the article is structured as follows: literature review and theoretical background (Section 2); material and methods (Section 3); results (Section 4); discussion (Section 5); conclusion, implications, and limitations (Section 6).

## 2 Literature review and theoretical background

### 2.1 Theoretical foundation

Drawing on social exchange theory (20, 21), intergenerational support can be conceptualized as a dynamic process in which tangible and intangible resources are exchanged within parent–child relationships. Such relationships can be sustained only when exchanges are perceived as reciprocal and fair, whereas imbalances may cause strain. Recent longitudinal research has provided empirical support for this theoretical framework by demonstrating that reciprocity operates through multiple pathways across the life course, including direct exchanges and indirect patterns transmitted across generations (73). From the perspective of older adults, support from adult children enhances the perception of reciprocity and fairness, thereby positively influencing psychological wellbeing. However, this positive effect may be contingent on how the support is interpreted. When support is viewed as an expected entitlement rooted in cultural norms rather than a voluntary act of exchange, its perceived reciprocity may diminish, potentially undermining its mental health benefits. In the Chinese context, research has shown that the balance of intergenerational exchanges matters more than the mere presence of support: older adults with imbalanced exchanges report poorer health outcomes, and both the direction and intensity of support flows significantly influence wellbeing (22). These findings highlight that the perceived fairness of exchanges, shaped by cultural expectations, is central to understanding how intergenerational support affects mental health in later life.

### 2.2 Child-to-parent intergenerational support and mental health

China embodies the traditional “foster-support” feedback model of familial relations, where parents raise their children, children in turn support their parents, and the cycle continues across generations as children raise their own offspring from whom they later receive support (23). In rural China, many older adults parents therefore live with their adult children and receive various forms of intergenerational support, including financial assistance, daily care, and emotional comfort. Where pension benefits are limited, particularly in rural areas, financial transfers from adult children play an important role by providing older adults parents with the means to withdraw from the labor market and meet daily needs. Such support fosters a sense of security and has been shown to have a positive effect on the mental wellbeing of older adults parents (24–26). Similar patterns have been observed in previous research on the impacts of daily care and emotional support. Bai and Gu (17), for instance, found that rural older adults who receive daily care from their children are more likely to report better self-rated health. Emotional support has likewise been shown to alleviate stress among older adults and reduce their risk of illness (27–30). Moreover, older adults individuals who receive greater levels of emotional support tend to evaluate their own health more positively (31, 32).

Despite this substantial evidence for the beneficial effects of intergenerational support, it is important to acknowledge that the literature is not entirely consistent. Some studies have reported mixed or null findings regarding the mental health consequences of support from adult children. For example, while Chen and Silverstein (14) found that financial and emotional support enhanced older parents’ subjective wellbeing, Zhang and Li (16) argued that reliance on children for financial assistance could adversely affect mental health. Similarly, Bai and Gu (17) identified mixed effects of instrumental and emotional support, suggesting that the relationship between support and mental health may be more complex than a simple positive association. The present study focuses on the potential positive effects of intergenerational support for two reasons. First, social support theory posits a main effect model in which support buffers against stress and promotes psychological wellbeing, providing a strong rationale for expecting beneficial effects. Second, in rural China where filial piety remains a deeply embedded social norm, support from adult children carries positive emotional significance and is interpreted as familial care rather than a threat to independence. Based on the above reasoning, the following hypothesis is thus proposed:

*Hypothesis 1*: Child-to-parent intergenerational support enhances the mental health of rural older adults.

### 2.3 Intergenerational emotional intimacy

Emotional intimacy refers to the perception of closeness to others, the sharing of personal feelings, and the experience of personal validation (33). It is a key dimension of the parent–child relationship, though its expression varies across cultures, family structures, and stages of the life course. While intimacy may be conveyed through both verbal and non-verbal communication, in Chinese society it is often expressed less through verbal affirmation and more through practical actions, such as financial support, caregiving, and co-residence (9, 24, 34–37). Studies have shown that receiving various forms of intergenerational support enhances the emotional connection felt by Chinese older adults toward their children (38). Older adults individuals who perceive greater intergenerational intimacy are more likely to engage in social interactions with their adult children and to report higher levels of mental health and wellbeing (14). Based on this, we propose that intergenerational emotional intimacy serves as a mediating mechanism linking support to mental health.

*Hypothesis 2*: Intergenerational support from children enhances the mental health of rural older adults parents by strengthening intergenerational emotional intimacy.

### 2.4 Traditional value

China is a society strongly shaped by Confucian culture. A core principle of Confucianism is filial piety (孝, xiao), which places a moral and social obligation on children to respect, obey, and care for their parents (39). This principle has been internalized in the national legal framework, such as the *Law on the Protection of the Rights and Interests of the Older Adults*, which stipulates that adult children have a duty to provide for and visit their parents (40). The principle is also imbedded in people’s everyday lives, for example, in social norms and moral standards (39, 41, 42). As a result, older adults parents may place significant importance on receiving filial piety from their children and often adhere to the traditional value of raising children for old-age support (43). However, one drawback of such adherence is that parents may develop elevated expectations regarding their children’s support. Disappointment is more likely to occur when such expectations are unmet, which can undermine intergenerational closeness and happiness (17). Although social norms and values surrounding old-age support are evolving in contemporary Chinese society, traditional norms remain prevalent among elder generations, particularly in rural and less-developed regions. Thus, we conceptualize traditional values as a moderating variable that conditions the relationship between support and emotional intimacy.

*Hypothesis 3*: The positive effect of intergenerational support from children on older adults parents’ perceived emotional intimacy with their children is weaker among those who adhere to the traditional value of raising children for old-age support.

**Figure 1:**
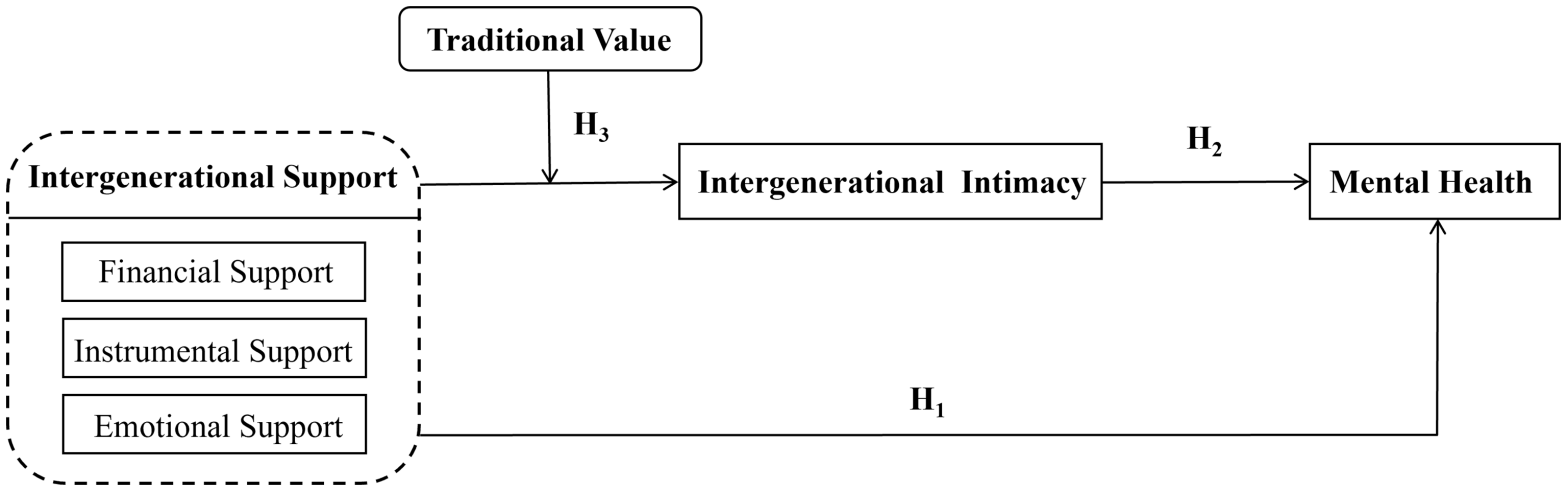
Research model.

Taken together, the three hypotheses presented above form an integrated conceptual framework, as illustrated in Figure 1. The model posits that intergenerational support affects rural older adults’ mental health through a mediated pathway conditioned by cultural values. Specifically, Hypothesis 1 proposes that child-to-parent intergenerational support (including financial, instrumental, and emotional support) directly enhances the mental health of rural older adults. Hypothesis 2 introduces intergenerational emotional intimacy as a mediating mechanism: intergenerational support improves mental health by strengthening the emotional bond between parents and children. Hypothesis 3 further specifies a moderating condition: the positive effect of support on emotional intimacy is weaker among older adults who strongly adhere to the traditional value of “raising children for old-age support.” In other words, when parents view children’s support as an expected obligation rooted in cultural norms rather than a voluntary expression of affection, the support is less likely to foster genuine emotional closeness, thereby diminishing its mental health benefits. This integrated framework thus captures not only the direct effects of support but also the psychological process (emotional intimacy) through which it operates and the cultural boundary condition (traditional values) that shapes its strength.

## 3 Materials and methods

### 3.1 Survey design

The questionnaire design was informed by previous research into mental health among older adults [e.g., see (29, 44)]. The final questionnaire covered the following aspects: First, we collected basic personal and family information, including age, gender, education, marriage and labor status (7), government subsidy, income, and offspring count. Second, we collected health-related information, including activities of daily living (ADL) (covering six activities: dressing, bathing, eating, getting in and out of bed, using the toilet, and controlling bowel and bladder movements), instrumental activities of daily living (IADL) (five activities: cooking, housekeeping, shopping, managing finances, and taking medication), depression scale (CES-D), This reverse coding was applied to facilitate interpretation of regression coefficients in relation to positive mental health outcomes, higher scores on the CESD-10 indicate better mental health (fewer depressive symptoms) (8, 45), chronic diseases, and medical expenses. Finally, intergenerational support was measured. This encompasses children’s place of residence, financial support (9) and daily life care provided by children, emotional closeness with children, following established research that has used contact frequency as a proxy for emotional closeness and support in large-scale surveys where direct measures of emotional support quality are unavailable (24, 35), we consider frequent contact to reflect ongoing emotional engagement and care. For each type of intergenerational support, financial support, instrumental support, and emotional support, higher scores indicate greater levels of support received from adult children. The definitions of all variables can be found in Table 1.

**Table 1:** Variable definitions.

Variables	Definition
Dependent variable	
Mental health	Measured by the 10-item Center for Epidemiologic Studies Depression Scale (CESD-10) developed by Andresen et al. (45). The scale includes ten items assessing depressive symptoms experienced during the past week: being bothered by things, trouble concentrating, feeling depressed, feeling that everything was an effort, feeling hopeful about the future, feeling fearful, restless sleep, feeling happy, feeling lonely, and being unable to get going. Respondents rated each item on a four-point scale ranging from 0 (rarely or none of the time) to 3 (most or all of the time). Following standard scoring procedures for the CESD-10, the two positive affect items (hopeful about the future and happy) were reverse-coded. Item scores were then summed to create a total score ranging from 0 to 30. In this study, higher scores on the CESD-10 indicate better mental health (fewer depressive symptoms). This reverse coding was applied to facilitate interpretation of regression coefficients in relation to positive mental health outcomes.
Life satisfaction	Measured using item: “Overall, how satisfied are you with your current life?” Respondents rated their satisfaction on a 5-point scale ranging from 1 (very dissatisfied) to 5 (very satisfied). Higher scores indicate greater life satisfaction.
Independent variables	
Intergenerational support	
Financial support	Annual financial transfers (including in-kind contributions) from adult children to older adults parents. 0 = none, 1 = 1–2,500, CNY, 2 = 2,501–5,000 CNY, 3 = 5,001–7,500 CNY, 4 = 7,501–10,000 CNY, 5 = over 10,000 CNY
Instrumental support	Annual frequency of household assistance (e.g., cleaning) and personal care support provided by adult children to older adults parents. 0 = almost never, 1 = several times a year, 2 = at least once a month, 3 = at least once a week, 4 = every day
Emotional support	Annual frequency of contact, including both in-person and remote communication (e.g., phone, WeChat), between older adults parents and their adult children. 0 = almost never, 1 = several times a year, 2 = at least once a month, 3 = at least once a week, 4 = every day
Instrumental variables	
town_avg_emo_support_outside	The village-level average of intergenerational support (excluding the respondent’s own children).
PNC (Proximity to Nearest Children)	The current residence of the children. 0 = this household, 1 = this village, 2 = this township, 3 = this county, 4 = other districts/counties in this city, 5 = other cities in this province, 6 = other provinces, 7 = overseas
Mechanism variables	
Intergenerational intimacy	Closeness with your child. 1 = not close, 2 = somewhat close, 3 = very close
Traditional value	Believe in “raising children for old-age support.” 1 = yes, 0 = no
Control variables	
Demographic characteristics	
Age	Age (years)
Gender	1, if male; 0, female
Education	1 = primary school and below, 2 = junior high school, 3 = senior high school/technical secondary school, 4 = junior college, 5 = bachelor’s degree or above
Marriage	1, if married; 0, otherwise
Labor status	1 = currently engaged in work, 2 = reduced work involvement, 3 = no longer working
Household characteristics	
Government subsidy	1, if household obtained; 0, otherwise
Income	0 = no amount (CNY), 1 = less than 20,000 CNY, 2 = 20,000–40,000 CNY, 3 = 40,000–60,000 CNY, 4 = 60,000–80,000 CNY, 5 = 80,000–100,000 CNY, 6 = 100,000–120,000 CNY, 7 = more than 120,000 CNY
Offspring count	Number of offspring
Objective health status	
Medical expense	Value of household’s medical expenses (CNY), log
ADL	Six activities: dressing, taking a bath, eating, getting in and out of bed, using the toilet, and controlling urination and defecation. 0 = cannot complete, 1 = have difficulties and need help, 2 = it can still be completed despite difficulties, 3 = no difficulty. The scores for each activity are accumulated, with a value range of 0 to 18.
IADL	Five activities: cooking, doing housework, shopping, financial management and taking medicine. 0 = cannot complete, 1 = have difficulties and need help, 2 = it can still be completed despite difficulties, 3 = no difficulty. The score for each activity is accumulated, with a value range of 0 to 15.
Chronic disease	The number of chronic diseases (0–13)

In the income options, the range “20,000–40,000 CNY” includes 40,000 CNY; the same logic applies to the following ranges. ADL (activities of daily living) measures basic self-care capacity; IADL (instrumental activities of daily). PNC refers to the proximity to the nearest child.

This study aimed to use survey data collected from rural older adults to establish a comprehensive understanding of child-to-parent intergenerational support and how this influences older adults mental health in a Chinese rural context, while considering the differences among older adults across regions. To obtain accurate micro-level data required for this study, relevant experts were first consulted to review and evaluate the initial questionnaire template, after which the initial questionnaire was revised. Subsequently, from July 28 to 30, 2024, the author conducted a small-scale pilot survey in Qiandeng Town, Kunshan City, using the modified questionnaire. Based on the responses collected during the pilot survey, the credibility and validity of the data were assessed and analyzed. Further issues identified in the pilot survey were discussed with experts and scholars both within and outside the university, leading to another round of revisions to the questionnaire used in the pilot. This process helped finalize the questionnaire for use in the formal survey.

### 3.2 Data collection

This study selected Kunshan City in Jiangsu Province and Yudu County in Jiangxi Province as case study sites. Both Kunshan City and Yudu County have demonstrated innovation and possess practical experience in older adults care services and health policies for older adults, such as Kunshan’s “Aging in Place with Support” service model (46) and Yudu’s “One Old, One Young” Happiness Village model (47). However, differences between the two case study regions also exist. Jiangsu Province, as an economically developed region, is a population inflow area, while Jiangxi Province typifies the development level of central China and is a population outflow area. The economic disparities between the two may lead to variations in intergenerational support and the health of the older adults population. For instance, older adults in the economically developed population inflow area of Kunshan may have higher socioeconomic status, while those in the population outflow area of Yudu may face different family support structures due to out-migration of adult children. These contextual differences inform our comparative analyses of regional heterogeneity in intergenerational support and mental health outcomes.

The questionnaire was piloted with eight respondents from July 28 to 30, 2024 in Qiandeng Town, Kunshan City, and then administered to rural adults over 60 years old in Kunshan, Jiangsu (*n* = 431) and Yudu, Jiangxi (*n* = 544) during November and December 2024. A combination of stratified sampling and random sampling was adopted. In each township, two administrative villages with a higher level of older adults care service development and two with a lower level were selected, along with one older adults care institution. The sample included 431 rural older adults individuals in Kunshan City (from 28 villages across 7 townships and from 5 older adults care institutions), and 544 rural older adults individuals in Yudu County (from 92 villages across 23 townships and from 19 older adults care institutions). The survey employed a one-on-one questionnaire method, to mitigate social desirability bias, all interviewers received specialized training and conducted one-on-one interviews in neutral locations such as community activity rooms. Prior to each interview, respondents were explicitly informed that there were no right or wrong answers, that the data would be used solely for academic research and kept strictly confidential, and that their children would not have access to the raw data. Furthermore, the CES-D scale was administered through self-completion for literate older adults, or by having the interviewer read each item aloud while the respondent selected answers independently, thereby minimizing the impact of third-party presence to the greatest extent possible. A total of 1,076 questionnaires were distributed, and 1,058 responses were collected, resulting in a questionnaire recovery rate of 98.32%. After excluding invalid questionnaires with issues such as unanswered questions, omissions, and logical errors in responses, 975 valid questionnaires were ultimately obtained, yielding an effective questionnaire rate of 92.16%.

### 3.3 Data analysis

#### 3.3.1 Benchmark empirical approach: OLS model

The model in this study was constructed as follows:

$$\begin{array}{l} \;{\text{Mhealth}}_{i}=\beta_{0}+\beta_{1}\;{\text{Fsupport}}_{i}+\beta_{2}\;{\text{Isupport}}_{i}\\ +\beta_{3}\;{\text{Esupport}}_{i}+\beta_{4}X_{i}+\varphi_{c}+\varepsilon_{i} \end{array}$$

where i is the individual, c is the county, and the dependent variable Mhealth represents mental health. Fsupport denotes financial support, Isupport denotes instrumental support, and Esupport denotes emotional support. Since mental health is measured as a continuous variable, ordinary least squares (OLS) regression is employed for estimation. *β_0_* represents the constant term, $X_{i}$ denotes a vector of control variables, $\varphi_{c}$ is the region dummy variable, and $\varepsilon_{i}$ is the error term.

#### 3.3.2 Identification strategy: IV strategies

Identifying the impact of intergenerational support on the mental health of the older adults may present the following challenges: First, reverse causality. The poorer the mental health of older adults parents, the more concerned their children are likely to be, which in turn leads to an increase in their financial support, daily care, and emotional support for their parents. Second, omitted variables. Although this study controls for numerous characteristics of older adults at the individual, family, and regional levels, there are many more factors influencing the mental health of older adults parents, making it difficult to incorporate all influencing factors into the model. Third, measurement error. The data in this study were collected through surveys conducted by the lead author, with all interviewers undergoing training and a pilot survey before the formal investigation. However, there may still be inaccuracies in interviewer records or respondent answers. Therefore, endogeneity is a key concern in the empirical study of the impact of child-to-parent intergenerational support on the mental health of rural older adults.

To address the issue of endogeneity, instrumental variables (IVs) at both the village and individual levels were identified. The village-level average of intergenerational support (excluding the respondent’s own children) (36) and the Proximity to Nearest Child (PNC) (48) are used as instrumental variables in our study. An instrumental variable must satisfy the relevance and exogeneity conditions. Regarding relevance, a “demonstration effect” is prevalent in rural China (49, 50), meaning that a household’s child-to-parent intergenerational support is significantly influenced by the support behaviors of other village households. The higher the village-level average of child-to-parent intergenerational support, the more likely a household’s children are to provide greater support, thus meeting the relevance requirement. Concerning exogeneity, the village-level average of intergenerational support (excluding the respondent’s own children) generally does not directly affect the health status of the older adults in the household (51), and the PNC is unlikely to have a direct effect on older adults parents’ mental health, except through the influence of this variable on the intergenerational support from the parents’ children (52). This method enables us to isolate the direct effect of the child-to-parent intergenerational support on the level of mental health in rural older adults households. We do this by running a two-stage instrumental variable strategy (2SLS-IV) (10). The following model is proposed to be established:

$$\begin{array}{c} \;{\text{Support}}_{j}=\beta_{o}+\beta_{1}IV_{i}+X_{i}+\mu_{i}+\varepsilon_{i}\\ \;{\text{Mhealth}}_{i}=\alpha_{o}+\alpha_{1}\;{\text{Support}}_{j}+X_{i}+\mu_{i}+\varepsilon_{i} \end{array}$$

Where *i* is the individual, j is the different dimensions of child-to-parent intergenerational support.

#### 3.3.3 Mediation analysis

To examine the mediating role of intergenerational emotional intimacy in the relationship between intergenerational support and mental health among rural older adults, we employed the two-step mediation approach proposed by Jiang (53). The procedure consists of two steps. First, estimate the association between intergenerational support and mental health using the regression models described in Section 3.4.1. This provides the total association. Second, estimate the association between intergenerational support and the proposed mediator, intergenerational emotional intimacy, using the same set of control variables. This indicates whether support is associated with the mediator. The mediator-outcome link should be grounded in theory rather than estimated from the same data. Therefore, if both associations are statistically significant, it is consistent with the hypothesis that emotional intimacy plays a mediating role. Following Jiang (53), we do not include the mediator in the mental health regression to avoid potential bias, nor do we formally decompose the total association into direct and indirect components. All results are interpreted as correlational patterns, not causal effects. The analysis was performed using Stata 17.0.

## 4 Results

### 4.1 Descriptive analysis and comparisons between regions

As noted in Section 3.2, the two samples exhibit distinct socio-demographic profiles consistent with their regional contexts. Table 2 presents a summary of the variables used in this study, which includes 975 observations. Overall, the means of the selected variables differ between Kunshan and Yudu. In comparison to rural older adults in Yudu, rural older adults in Kunshan are typically more educated, more likely to be married and engaged in employment, and have larger offspring counts and higher incomes. They also report lower medical expenses and less chronic disease, and receive higher instrumental support and emotional support, but lower financial support. These differences indicate that the impact of intergenerational support on the mental health of rural parents may vary across regions, which should be addressed in the empirical analysis.

**Table 2:** Descriptive statistics.

Variables	Total		Kunshan		Yudu	
	Mean/%	SD	Mean/%	SD	Mean/%	SD
Dependent variable						
Mental health	23.810	5.685	26.300	4.422	21.830	5.799
Life satisfaction	3.798	0.826	4.070	0.689	3.583	0.863
Independent variables						
Intergenerational support						
Financial support	1.548	1.443	1.476	1.601	1.605	1.303
Instrumental support	1.763	1.491	1.921	1.561	1.638	1.422
Emotional support	2.881	1.062	3.348	0.783	2.511	1.107
Instrumental variables						
town_avg_emo_support_outside	2.881	0.494	3.348	0.0810	2.511	0.351
PNC	2.319	1.840	1.956	1.491	2.607	2.031
Mechanism variables						
Intergenerational intimacy	1.772	0.470	1.884	0.335	1.684	0.539
Traditional value (% believe in “raising children for old-age support”)	83.3	—	81.0	—	85.1	—
Control variables						
Demographic characteristics						
Age	71.20	7.166	70.83	6.797	71.48	7.438
Gender (% male)	61.9	—	64.5	—	59.9	—
Education	1.506	0.743	1.596	0.795	1.434	0.691
Marriage (% married)	78.6	—	0.879	—	0.711	—
Labor status						
Currently engaged in work (%)	26.3	—	27.4	—	25.4	—
Reduced work involvement (%)	26.0	—	21.8	—	29.4	—
No longer working (%)	47.7	—	50.8	—	45.2	—
Household characteristics						
Government subsidy (% household obtained)	9.2	—	6.5	—	11.4	—
Income	1.906	1.722	3.350	1.494	0.761	0.762
Offspring count	2.446	1.377	1.585	0.782	3.129	1.365
Objective health status						
Medical expense (log)	4.584	5.575	4.035	5.989	5.018	5.188
ADL	16.72	1.733	15.770	1.093	17.480	1.773
IADL	13.80	2.631	14.48	1.882	13.25	2.990
Chronic disease	1.407	1.251	1.262	1.175	1.522	1.297

Continuous variables are summarized as means (with three decimal places) and standard deviations, binary variables (coded 0/1) are presented as percentages. ADL (activities of daily living) measures basic self-care capacity; IADL (instrumental activities of daily). PNC refers to the proximity to the nearest child. town_avg_emo_support_outside refers to the village-level average of emotional support, excluding the respondent’s own children.

The internal consistency reliability of the CES-D scale was evaluated using Cronbach’s alpha. The analysis yielded a coefficient of 0.8389, suggesting excellent scale reliability and robust consistency among the items, thus supporting the stability and dependability of the measurement tool.

Disparities were observed in Kunshan and Yudu in terms of rural older adults mental health status. Specifically, older adults in Kunshan exhibited a mean CES-D score of 26.30 (SD = 4.42), which is 2.49 points higher than the full sample mean of 23.81, indicating better mental health in this region. In contrast, Yudu’s rural older adults had a mean CES-D score of 21.83 (SD = 5.80), 1.98 points below the full sample average, suggesting a more severe depression burden in this area.

The multidimensional patterns of child-to-parent intergenerational support showing a clear hierarchy: emotional support (2.881) > instrumental support (1.763) > financial support (1.548). This suggests that emotional exchanges are the most prevalent form of intergenerational support, reflecting stronger emotional bonds, while financial and practical care support remain relatively limited. Regional comparisons reveal that Kunshan provides higher overall support, particularly in instrumental care and emotional support. However, Yudu exhibits slightly greater financial assistance, suggesting that in less-developed regions, weaker institutional support maintains greater reliance on traditional financial transfers, whereas developed regions like Kunshan have transitioned toward emotion-based intergenerational solidarity supported by more comprehensive social security systems.

With regard to intergenerational intimacy and the traditional value of raising children for support in old age, the average intergenerational intimacy in Kunshan is higher than that in Yudu, while adherence to this value is lower than in Yudu. To some extent, this indicates that in economically developed areas (such as Kunshan), intergenerational emotional ties are closer, family values may be more modern, individual independence is enhanced, and traditional values are relatively diluted. In less economically developed areas (such as Yudu), children may migrate for work, leading to greater physical distance and relatively lower emotional intimacy. These areas are less impacted by modernization and external cultural influences, thus retaining more traditional family values such as raising children for old-age support.

### 4.2 Effect of intergenerational support on mental health

#### 4.2.1 Estimation results

To examine the effects of intergenerational support on mental health, we conducted a hierarchical regression analysis (Table 3). Multicollinearity diagnostics revealed no serious concerns, with a mean VIF of 1.63 and all individual VIF values below 5.0, indicating no serious multicollinearity concerns. Table 3 reports the empirical results examining the effects of intergenerational support from children on the mental health of rural older parents. The analysis employs a hierarchical regression approach: column (1) includes only the core explanatory variables, columns (2), (3), and (4) sequentially incorporate demographic characteristics, objective health status, and household characteristics, and column (5) adds regional fixed effects. The model fit statistics at the bottom of Table 3 show the change in *R*^2^ (Δ*R*^2^) at each step. Adding demographic characteristics in Model 2 increased *R*^2^ by 0.069, which was statistically significant (*p* < 0.001). The inclusion of health status in Model 3 led to a much larger increase of 0.204 (*p* < 0.001), indicating that health status explains a substantial portion of the variance in mental health. Adding household characteristics in Model 4 produced a small and non-significant increase (Δ*R*^2^ = 0.019, *p* = 0.111). Finally, including regional fixed effects in Model 5 increased *R*^2^ by 0.012, which was statistically significant (*p* < 0.001). Overall, demographic and health factors are the strongest contributors to the explained variance in mental health.

**Table 3:** Benchmark results: OLS regression of intergenerational support on mental health.

Mental health	(1)	(2)	(3)	(4)	(5)
Financial support	0.0552	0.0808	0.1540	0.1600	0.1819^*^
	(0.1146)	(0.1136)	(0.1048)	(0.1004)	(0.0987)
Instrumental support	0.0093	0.0303	0.0539	0.0590	0.0476
	(0.1175)	(0.1164)	(0.1046)	(0.0996)	(0.0989)
Emotional support	1.3941^***^	1.3148^***^	0.9372^***^	0.8066^***^	0.6427^***^
	(0.1778)	(0.1780)	(0.1650)	(0.1589)	(0.1601)
Age		0.0877^***^	0.1068^***^	0.0936^***^	0.0716^**^
		(0.0271)	(0.0263)	(0.0288)	(0.0288)
Gender		1.1938^***^	0.9542^***^	0.9227^***^	1.0004^***^
		(0.3886)	(0.3602)	(0.3456)	(0.3419)
Education		0.4639^*^	0.4607^**^	0.3337	0.3460
		(0.2425)	(0.2297)	(0.2213)	(0.2189)
Marriage		1.9738^***^	1.4917^***^	1.0799^**^	1.0494^**^
		(0.4983)	(0.4594)	(0.4584)	(0.4571)
Labor status		−1.1115^***^	−0.5918^***^	−0.3545^*^	−0.4294^**^
		(0.2072)	(0.1939)	(0.1912)	(0.1917)
Medical expense			−0.1641^***^	−0.1251^***^	−0.1190^***^
			(0.0272)	(0.0270)	(0.0269)
ADL			−0.4765^***^	−0.1962	0.0386
			(0.1408)	(0.1428)	(0.1438)
IADL			0.8835^***^	0.6239^***^	0.5198^***^
			(0.0829)	(0.0843)	(0.0889)
Chronic disease				−1.0419^***^	−0.9983^***^
				(0.1347)	(0.1328)
Government subsidy				0.1494	0.2975
				(0.6022)	(0.5886)
Income				0.5526^***^	0.2382^**^
				(0.1086)	(0.1198)
Offspring count				−0.0204	0.2201
				(0.1473)	(0.1549)
County FE					−2.5172^***^
					(0.5579)
*N*	975	975	975	975	975
adj. *R*^2^	0.066	0.131	0.289	0.350	0.362
Δ*R*^2^		0.069	0.204	0.019	0.012
Test statistic		6.42	50.79	2.01	20.36
*p*-value		0.0000	0.0000	0.1106	0.0000

Δ*R*^2^ represents the change in *R*^2^ from the previous model. The significance of Δ*R*^2^ was assessed using robust Wald tests for the joint significance of the added variable block (tested in the final model). Degrees of freedom are based on the residual degrees of freedom of the final model (df = 958). ADL (activities of daily living) measures basic self-care capacity; IADL (instrumental activities of daily living) measures more complex functional abilities. Unstandardized coefficients are reported with standard errors in parentheses. Column 1 includes only core explanatory variables (financial, instrumental, and emotional support). Column 2 adds demographic controls (age, gender, education, marriage, labor status). Column 3 adds objective health status (medical expense, ADL, IADL, chronic disease). Column 4 adds household characteristics (government subsidy, income, offspring count). Column 5 adds regional fixed effects. ^***^, ^**^, and ^*^ indicate significance at the 1, 5, and 10% levels, respectively.

The regression coefficients for emotional support are statistically significant at the 1% level across all columns, indicating that emotional support constitutes a critical factor in maintaining the mental health of the older adults. After incorporating control variables, the regression coefficient of emotional support decreased from 1.3941 to 0.6427, remaining statistically significant at the 1% level. This indicates that, with other factors remaining unchanged, each one-unit increase in emotional support from children is associated with an average increase of 0.6427 points in mental health scores of the older adults. Financial support only achieved statistical significance after incorporating regional fixed effects, indicating that its positive effect on mental health of rural older adults is mediated by local economic conditions, while the regression coefficient instrumental support failed to show statistical significance. The first research hypothesis of child-to-parent intergenerational support enhances the mental health of rural older adults has therefore been partially validated.

#### 4.2.2 Treatment of endogenous variables—instrumental variable analysis

To address potential endogeneity, we conducted an instrumental variable (IV) regression using the two-stage least squares (2SLS) method. As shown in Table 4, the LM statistic of the unidentifiable test is 36.917, with a corresponding *p*-value of 0.000, indicating that the selected instrumental variable is identifiable. The Wald statistic of the weak instrumental variable test is 24.182, which is greater than the Stock-Yogo critical value at the 10% significance level, indicating that the instrumental variable has good explanatory power for endogenous variables. Meanwhile, the *p*-value corresponding to the Hansen-j statistic is greater than 0.1, proving that there is no problem of overidentification. From the estimation results of the first stage, the regression coefficients of the instrumental variables are all significantly positive, confirming a strong correlation between the instrumental variables and emotional support. The estimation results in the second column show that the coefficient of emotional support remains robustly positive, with its absolute value increasing compared to the baseline regression. This further demonstrates that, after partially controlling for endogeneity issues caused by omitted variable bias and reverse causality, emotional support from children still exerts a significantly positive effect on the mental health of rural older adults, confirming the robustness of our benchmark regression results.

**Table 4:** Instrumental variable analysis: two-stage least squares (2SLS) estimates.

Variables	The first phase	The second phase
	s_Emotional support	Mental health
town_avg_emo_support_outside	0.5864^***^	
	(0.1393)	
PNC	−0.0849^***^	
	(0.0181)	
s_Emotional support		1.6839^**^
		(0.6887)
Controls	Yes	Yes
County FE	Yes	Yes
Phase 1 value	19.97	
*Underidentification test*		36.917 (0.000)
*Weak identification test*		24.182 [19.93]
*Overidentification test*		1.763 (0.1843)
*Centered R^2^*		0.341
*Uncentered R^2^*		0.965
*N*		975

The s_Emotional support represents the fitted value of emotional support, derived using instrumental variables along with other exogenous variables. PNC refers to the proximity to the nearest child. town_avg_emo_support_outside refers to the village-level average of emotional support, excluding the respondent’s own children. Unstandardized coefficients are reported with standard errors in parentheses. ^***^, ^**^, and ^*^ indicate significance at the 1, 5, and 10% levels, respectively.

#### 4.2.3 Robustness checks

Replacement of the explained variable: Life satisfaction is another commonly used indicator for measuring mental health in related studies (5). The estimation results in column (1) of Table 5 show that higher levels of financial support and emotional support from children are associated with greater parental life satisfaction. These empirical findings further demonstrate that financial and emotional support from children contribute to improving parents’ mental health, confirming the robustness of the baseline regression conclusions.

**Table 5:** Robustness checks.

Variables	(1)	(2)	(3)
	Life satisfaction	Depression	Mental health
Financial support	0.1456^***^	0.1996	0.1474
	(0.0449)	(0.1468)	(0.1001)
Instrumental support	−0.0047	0.1394	0.0365
	(0.0457)	(0.1307)	(0.1003)
Emotional support	0.2036^***^	0.2772^*^	0.6990^***^
	(0.0687)	(0.1459)	(0.1596)
Controls	Yes	Yes	Yes
County FE	Yes	Yes	Yes
*N*	975	975	936
Pseudo *R*^2^/*R*^2^	0.098	0.551	0.346
adj. *R*^2^			0.335

Standard errors are reported in parentheses. ^***^, ^**^, and ^*^ indicate significance at the 1, 5, and 10% levels, respectively. In columns (1) and (2), the dependent variable is categorical. Ordered logistic (ologit) models are estimated; because these are nonlinear regressions, Pseudo *R*^2^ is reported. In column (3), an ordinary least squares (OLS) model is estimated, and the standard *R*^2^ is reported.

Alternative measures of mental health: To test whether the main findings are sensitive to the operationalization of mental health, we replaced the continuous CES-D score with a binary indicator of depressive symptoms. Following Andresen et al. (45), a CES-D score greater than 10 is considered indicative of depressive symptoms. Reverse scoring was used in this study, where a mental health score below 20 indicates depressive symptoms. In our measurement, a mental health score below 20 was coded as 0 (depressed), otherwise 1. The results in column (2) of Table 5 show that emotional support helps alleviate depressive symptoms among rural older adults parents, confirming the findings of the baseline regression.

Replacement of the sample: To assess whether the results are driven by the most vulnerable older adults, we conducted a subsample analysis excluding respondents with significant functional limitations. Older adults with difficulties in one or more activities of daily living (ADLs) were classified as disabled older adults, resulting in the exclusion of 39 disabled samples from the analysis. As shown in column (3) of Table 5, after excluding disabled samples, the coefficient of emotional support on rural older adults’s mental health remains statistically significant at the 1% level with a value of 0.6990, while the coefficients of financial support and instrumental support are insignificant. This further demonstrates the reliability and robustness of our research findings.

### 4.3 Moderated mediation

This section examines intergenerational intimacy as mediating variables and adherence to the traditional value of raising children for old-age support as moderating variables, with the results presented in Table 6.

**Table 6:** Moderated mediation: the role of intergenerational intimacy and traditional values.

Variables	(1)	(2)
	Intergenerational intimacy	Intergenerational intimacy
Financial support	0.2063^***^	0.0696^***^
	(0.0742)	(0.0199)
Instrumental support	0.0774	0.0177
	(0.0639)	(0.0313)
Emotional support	0.4446^***^	0.2035^***^
	(0.0896)	(0.0474)
Traditional value		0.6560^***^
		(0.1537)
Financial support × traditional value		−0.0529^**^
		(0.0218)
Instrumental support × traditional value		−0.0049
		(0.0326)
Emotional support × traditional value		−0.1473^***^
		(0.0492)
Controls	Yes	Yes
County FE	Yes	Yes
*N*	975	975
*R^2^*	0.116	0.187

Unstandardized coefficients are reported with standard errors in parentheses. ^***^, ^**^, and ^*^ indicate significance at the 1, 5, and 10% levels, respectively.

Column (1) presents the first step of the mediation analysis following Jiang (53), regressing intergenerational intimacy on intergenerational support, holding other factors constant, for every one-level increase in the financial support provided by children to their parents, the average score of intergenerational intimacy rises by 0.2063 points, an indication that financial support significantly enhances intergenerational intimacy overall. When the frequency of visits or contact between children and parents increases by one level, the average score of intergenerational intimacy improves by 0.4446 points, suggesting that emotional support, fulfilled through frequent interaction, meets core emotional needs and exerts a stronger reinforcing effect on intergenerational intimacy. Following the analytical strategy recommended by Jiang (53), we do not include the mediator in the mental health regression to avoid potential bias. Instead, we rely on existing literature to support the link from intergenerational intimacy to mental health. Consistent with previous research findings (54–56), intergenerational intimacy focuses on family cohesion and is regarded as a crucial component of family relationships, exerting significant influence on individuals’ psychological wellbeing across all stages of the life course. The enhancement of intergenerational intimacy helps older adults cope with stress, and promotes healthy aging and social integration, thereby playing a vital role in the mental health of rural older adults (29, 57). Taken together, these patterns are consistent with Hypothesis 2, which posits that intergenerational support is associated with better mental health through its positive association with intergenerational emotional intimacy.

Column (2) examines the moderating effect of traditional values on the relationship between intergenerational support and mental health using an interaction term, it can be observed that financial support from children and its interaction with the traditional value of raising children for support in old age is significantly correlated with intergenerational intimacy, as is emotional support from children and its interaction with this value. Specifically, for older adults individuals who adhere to this value, the positive effect of each additional unit of financial support from children on intergenerational intimacy is 0.0529 units smaller than for those who do not. Similarly, each additional unit of emotional support from children is associated with a 0.1473-unit smaller increase in intergenerational intimacy for those holding this traditional view. These results provide evidence that adherence to the value of raising children for old-age support acts as a negative moderator in the relationship between support from children and intergenerational intimacy. Rather than strengthening the positive effects of support, it dilutes them, with a particularly pronounced dilution effect on emotional support. Hypothesis 3, which posits that the positive effect of intergenerational support on emotional intimacy is weaker among older adults who adhere to the traditional value of raising children for old-age support, has therefore also been partially validated.

### 4.4 Heterogeneity analysis

#### 4.4.1 Region

Table 7 investigated whether the effects of intergenerational support differ across regions by running separate regressions for Kunshan and Yudu subsamples. Specifically, in Kunshan, intergenerational support shows no statistically significant impact on older adults mental health, whereas in Yudu, both financial support and emotional support exhibit significantly positive effects. These regional variations demonstrate that the effects of financial and emotional support are highly dependent on socioeconomic contexts. In less-developed rural areas like Yudu, financial and emotional support from children serves as a cornerstone for older adults mental health. However, in more developed regions such as Kunshan, the role of child-to-parent intergenerational support in older adults health appears to be substituted by other modernization factors.

**Table 7:** Intergenerational support and older adults mental health in rural China: heterogeneity analysis by region.

Variables	(1)	(2)
	Kunshan	Yudu
Financial support	0.0567	0.3076^**^
	(0.1302)	(0.1549)
Instrumental support	−0.0602	0.0587
	(0.1228)	(0.1572)
Emotional support	0.0080	0.8931^***^
	(0.2431)	(0.2021)
Controls	Yes	Yes
County FE	Yes	Yes
*N*	431	544
adj. *R*^2^	0.155	0.312
*R* ^2^	0.184	0.331

Unstandardized coefficients are reported with standard errors in parentheses. ^***^, ^**^, and ^*^ indicate significance at the 1, 5, and 10% levels, respectively.

#### 4.4.2 Gender

To explore gender differences, we split the sample by sex and re-estimated the main model (Table 8). There are inherent psychological differences between fathers and mothers, with mothers being more emotionally vulnerable and more attuned to emotional feedback than fathers (58). Contrary to existing studies, Table 8 shows that emotional support has significant positive effects on the mental health for both male and female respondents, with a larger coefficient for males (0.7757, *p* < 0.01) than for females (0.4332, *p* < 0.05). Financial support was significantly associated with better mental health only among females (0.2829, *p* < 0.05); no significant effect was found for males. Instrumental support was not significant for either gender.

**Table 8:** Intergenerational support and older adults mental health in rural China: heterogeneity analysis by gender.

Variables	(1)	(2)
	Male	Female
Financial support	0.0942	0.2829^*^
	(0.1264)	(0.1610)
Instrumental support	−0.0030	0.1487
	(0.1169)	(0.1678)
Emotional support	0.7757^***^	0.4332^*^
	(0.1984)	(0.2444)
Controls	Yes	Yes
County FE	Yes	Yes
*N*	604	371
adj. *R*^2^	0.396	0.317
*R* ^2^	0.411	0.345

Unstandardized coefficients are reported with standard errors in parentheses. ^***^, ^**^, and ^*^ indicate significance at the 1, 5, and 10% levels, respectively.

#### 4.4.3 Age

The advancement of age in older adults is often accompanied by the decline of physical functions, which in turn is closely linked to mental health. Therefore, the impact of intergenerational support on the mental health of older adults may vary across different age groups.

To capture age-related differences, we re-estimated the main model separately for three age groups: 60–69, 70–79, and 80 years or older (Table 9). As shown in Table 9, the influence of emotional support on the mental health of rural older adults diminishes with age. The effect is most pronounced among those aged 60–69, whereas no significant impact is observed in the group aged 80 and above. For early older adults (aged 60–69), who have just entered old age, the primary need is psychological adaptation. At this stage, emotional support from children helps parents adapt to social role transitions and provides psychological comfort. For middle older adults (aged 70–79), physical functions decline, mobility decreases, and the demand for medical and older adults care facilities increases significantly. The effect of emotional support is diluted by health concerns. For late older adults (aged 80+), severe physical decline, reduced cognitive ability, or extremely diminished social circles—even semi-disability or disability—may hinder the transmission of emotional support. The ability of people in this age group to perceive emotions diminishes, making them more reliant on basic daily care, though this effect is not statistically significant in the model.

**Table 9:** Intergenerational support and older adults mental health in rural China: heterogeneity analysis by age.

Variables	(1)	(2)	(3)
	60 < Age < 69	70 < Age < 79	Age> = 80
Financial support	0.1220	0.2154	0.1141
	(0.1448)	(0.1506)	(0.3267)
Instrumental support	0.1598	−0.0424	0.1382
	(0.1494)	(0.1385)	(0.3571)
Emotional support	0.8880^***^	0.4431^**^	0.1910
	(0.2579)	(0.2194)	(0.4851)
Controls	Yes	Yes	Yes
County FE	Yes	Yes	Yes
*N*	412	438	125
adj. *R*^2^	0.410	0.408	0.119
*R* ^2^	0.433	0.430	0.233

Unstandardized coefficients are reported with standard errors in parentheses. ^***^, ^**^, and ^*^ indicate significance at the 1, 5, and 10% levels, respectively.

#### 4.4.4 Family structure

We also examined whether family structure (single-child vs. multi-child) conditions the observed relationships, as shown in Table 10, the impact of child-to-parent intergenerational support on rural older adults’s mental health exhibits distinct family structure heterogeneity. Both financial and emotional support from children demonstrate significantly positive effects on mental health for older adults parents with multiple children, whereas in single-child families, child-to-parent intergenerational support shows no statistically significant impact on older adults mental health. This pattern may be explained by two factors: First, multiple children enable “risk diversification” in eldercare, where siblings can complement each other by sharing financial responsibilities and dividing emotional companionship duties, thereby improving older adults mental health. Second, under the traditional Chinese rural belief that “more children mean more blessings”, having multiple children itself serves as a psychological comfort source. In contrast, the non-significant findings in single-child families may reflect the unique psychological pressures faced by older parents in rural China. For these parents, having only one child means concentrating all filial expectations on a single individual. This creates a psychological dilemma: they depend entirely on that child for support, yet simultaneously worry about overburdening them. Such concerns may offset the mental health benefits of whatever support is received.

**Table 10:** Intergenerational support and older adults mental health in rural China: heterogeneity analysis by family structure.

Variables	(1)	(2)
	Single-child family	Multiple-child family
Financial support	−0.1034	0.1819^*^
	(0.1610)	(0.0987)
Instrumental support	−0.0942	0.0476
	(0.1623)	(0.0989)
Emotional support	0.3524	0.6427^***^
	(0.3332)	(0.1601)
Controls	Yes	Yes
County FE	Yes	Yes
*N*	274	975
adj. *R*^2^	0.334	0.362
*R* ^2^	0.371	0.373

Unstandardized coefficients are reported with standard errors in parentheses. ^***^, ^**^, and ^*^ indicate significance at the 1, 5, and 10% levels, respectively.

## 5 Discussion

The results showed that overall, child-to-parent intergenerational support enhances the mental health of rural older adults in both case study regions, consistent with previous studies (27–32). However, the positive impact of financial and emotional support was observed only among respondents from Yudu. Such a difference by study region might relate to the varying economic status between the two locations. In less-developed areas (e.g., in Yudu), where economic pressures remain unmitigated and traditional family emotional bonds may be strained by out-migration, economic and emotional support become critical for safeguarding the mental health of the older adults population (59). In more developed areas, where economic and basic care needs are less of a challenge, well-established pension systems, health insurance, and community service systems may substitute for or dilute the marginal utility of support from adult children (24). Surprisingly, instrumental support showed no direct effect on the mental health of rural older adults. This may be because instrumental support is typically required only when older adults experience declining health and functional limitations. Such assistance may be perceived more as a necessary obligation rather than a pure expression of affection and might sometimes even undermine elders’ self-esteem (60), resulting in potentially limited direct benefits for psychological wellbeing.

Gender differences were observed in the impact of intergenerational support on the mental health of rural older adults. Specifically, economic support was influential only among women, whereas emotional support was more influential among men. Several mechanisms may explain this pattern. In traditional Chinese rural contexts, men are typically responsible for external affairs and serve as economic providers and primary decision-makers, while women are more often associated with domestic responsibilities (61). Moreover, Chinese men are less likely to express anxiety or loneliness and often suppress emotional expression over the long term, resulting in less frequent and less intimate daily emotional interactions with their children compared to women (62). As a result, emotional support from children may be particularly compensatory for men. In contrast, women have long assumed caregiving roles within the family and are more adept at maintaining emotional connections through mutual help in household chores and casual conversations with neighbors in rural society. Therefore, the incremental impact of additional emotional support from children is relatively weaker on women than on men. Furthermore, women typically have fewer opportunities to participate in agricultural production or non-agricultural employment and often hold disadvantaged positions in the distribution of family resources, such as land and pension entitlements (63). Consequently, economic support from children can directly alleviate women’s concerns about livelihood security, thereby improving their mental health. For instance, financial support may enhance women’s ability to engage in a greater variety of day-to-day activities or reduce feelings of being “house-bound,” thereby improving their psychological wellbeing (as suggested by the reviewer). It may also decrease stress related to financial concerns, particularly for women who have limited access to independent income sources.

The results show that most respondents adhere to the traditional value of raising children for old-age support, consistent with findings from previous studies (64–67). This value can be particularly beneficial for older adults in rural China, where pension provision is limited and the government-led old-age care system remains underdeveloped. However, it has also been found to weaken the positive effect of intergenerational support on the mental health of rural older adults in our study. One possible explanation lies in the distinction between support perceived as obligatory duty versus voluntary intimacy. When parents strongly endorse traditional filial values, they are more likely to view children’s support as a mandatory obligation rooted in cultural norms rather than an act of genuine affection. This obligation-based framing has two interrelated consequences. First, it instrumentalizes the parent–child relationship, framing it as a form of market-based economic exchange that demands precise and immediate reciprocity (68). Second, it elevates parents’ expectations regarding the amount and timing of support, leading them to take such support for granted rather than appreciating it as a discretionary gift (31). When support is perceived as “what children owe” rather than “what children willingly give,” its emotional significance is diminished (69, 70). Consequently, even when economic and emotional support is received, it becomes less likely to be transformed into genuine intergenerational intimacy, thereby weakening its positive impact on parents’ mental health (71).

## 6 Conclusion, implications, and limitations

This study highlights that intergenerational support, particularly economic and emotional support, plays an important role in enhancing the mental health of rural older adults, both directly and through the influence of such support on perceived intergenerational intimacy. However, adherence to the traditional value of raising children for old-age support weakens this positive effect, as it tends to instrumentalize parent–child relationships. Moreover, the impacts of intergenerational support on mental health vary across regions and between genders.

Based on the findings, several policy implications can be drawn. First, policies should be tailored to regional and gender differences rather than adopting a one-size-fits-all approach. In less-developed areas such as Yudu, where financial insecurity is more prevalent, policy priorities should focus on strengthening family-based older adults care functions. Measures could include raising the standard of rural pensions, expanding the coverage of the subsistence allowance system, and implementing industrial support initiatives to increase local employment and reduce the out-migration of young people. Tax incentives could further encourage children to provide financial support, while programs should be introduced to promote intergenerational emotional communication. In more developed areas such as Kunshan, where material needs are better met, the emphasis should shift toward diversified and professional social older adults care services. Expanding access to high-quality community-based and institutional care, while also encouraging older adults to extend their social networks beyond the family, can help meet their growing psychological and emotional needs as they age.

Second, attention to gender differences is critical. Since economic support has a stronger impact on women’s mental health, targeted subsidies and social assistance programs for rural women could help reduce economic insecurity. For men, emotional support is particularly influential; thus, platforms offering emotional counseling, as well as initiatives encouraging children to maintain regular, meaningful communication with their fathers, should be promoted. Third, as the core of rural older adults care is gradually shifting from material to emotional needs, policies must also address cultural values. Efforts should be made to encourage families and communities to recognize that emotional support is as important as material support. Public campaigns, such as the creation and dissemination of short videos on social media platforms, could help reshape traditional expectations of “raising children for old-age support” and promote new norms of intergenerational intimacy and shared responsibility. Overall, local governments should carefully assess the actual demands of rural older adults, align resource allocation with the level of regional development, and create supportive conditions that enable younger generations to provide both economic and emotional support. By doing so, policies can better sustain the wellbeing of older adults while ensuring intergenerational harmony in rural China.

This study has several limitations. First, due to the cross-sectional research design, the data only reflect a snapshot at a single point in time and cannot capture the dynamic processes of change between intergenerational support and mental health. In the context of rural families, factors such as the cycles of adult children migrating for work, the health status of the older adults, and family relations are in constant flux. These changes can simultaneously affect the provision of intergenerational support and the psychological state of older adults. Therefore, the correlations identified in this study cannot be interpreted as strict causal relationships. Future research could consider employing longitudinal data to further explore the dynamic associations between intergenerational support and mental health over time. Another limitation of this study stems from the data collection method. Both mental health (measured by the CES-D scale) and support frequency were assessed through self-reports, which may introduce recall bias and social desirability bias, particularly when supportive behaviors are part of daily routines and older adults in rural contexts may overreport children’s support or underreport depressive symptoms to maintain family reputation. Future research could attempt to combine data from multiple sources for cross-validation, such as incorporating reports from adult children or objective observations. In addition, the measurement of instrumental support in this study has certain limitations. Due to the constraints of the questionnaire design, we were only able to capture the overall frequency of instrumental support, rather than distinguishing between more specific subtypes such as household assistance and sickness care. In this study, the non-significant findings for instrumental support in certain models may stem from this heterogeneity being masked by the aggregate measurement. For instance, the protective effect of sickness care might be offset by other types of support that show no significant association with mental health. Future research should employ more refined measurement tools to examine the differential effects of various types of instrumental support on the mental health of rural older adults. Finally, this study surveyed older adults individuals only in Kunshan, Jiangsu, and Yudu, Jiangxi. Caution is needed when generalizing the conclusions to broader populations. Future research could select more diverse samples across the country to confirm the generalizability of the findings.

## Data Availability

The original contributions presented in the study are included in the article/Supplementary material, further inquiries can be directed to the corresponding author.
